# Saxitoxin Puffer Fish Poisoning in the United States, with the First Report of *Pyrodinium bahamense* as the Putative Toxin Source

**DOI:** 10.1289/ehp.8998

**Published:** 2006-07-06

**Authors:** Jan H. Landsberg, Sherwood Hall, Jan N. Johannessen, Kevin D. White, Stephen M. Conrad, Jay P. Abbott, Leanne J. Flewelling, R. William Richardson, Robert W. Dickey, Edward L.E. Jester, Stacey M. Etheridge, Jonathan R. Deeds, Frances M. Van Dolah, Tod A. Leighfield, Yinglin Zou, Clarke G. Beaudry, Ronald A. Benner, Patricia L. Rogers, Paula S. Scott, Kenji Kawabata, Jennifer L. Wolny, Karen A. Steidinger

**Affiliations:** 1 Fish and Wildlife Research Institute, Florida Fish and Wildlife Conservation Commission, St. Petersburg, Florida, USA; 2 Food and Drug Administration, Center for Food Safety and Applied Nutrition, Laurel, Maryland, USA; 3 Food and Drug Administration, Office of the Commissioner, Rockville, Maryland, USA; 4 Food and Drug Administration, Center for Food Safety and Applied Nutrition, College Park, Maryland, USA; 5 Food and Drug Administration, Center for Food Safety and Applied Nutrition, Gulf Coast Seafood Laboratory, Dauphin Island, Alabama, USA; 6 National Oceanic and Atmospheric Administration, National Ocean Service, Center for Coastal Environmental Health and Biomolecular Research, Charleston, South Carolina, USA; 7 Key Laboratory of Science and Engineering for Marine Ecology and Environment, First Institute of Oceanography, State Oceanic Administration, Qingdao, China; 8 Florida Institute of Oceanography, University of South Florida, St. Petersburg, Florida, USA

**Keywords:** dinoflagellate, Florida, harmful algae, puffer fish, *Pyrodinium bahamense*, saxitoxin puffer fish poisoning, saxitoxins, *Sphoeroides* spp

## Abstract

**Background:**

From January 2002 to May 2004, 28 puffer fish poisoning (PFP) cases in Florida, New Jersey, Virginia, and New York were linked to the Indian River Lagoon (IRL) in Florida. Saxitoxins (STXs) of unknown source were first identified in fillet remnants from a New Jersey PFP case in 2002.

**Methods:**

We used the standard mouse bioassay (MBA), receptor binding assay (RBA), mouse neuroblastoma cytotoxicity assay (MNCA), Ridascreen ELISA, MIST Alert assay, HPLC, and liquid chromatography-mass spectrometry (LC-MS) to determine the presence of STX, decarbamoyl STX (dc-STX), and *N*-sulfocarbamoyl (B1) toxin in puffer fish tissues, clonal cultures, and natural bloom samples of *Pyrodinium bahamense* from the IRL.

**Results:**

We found STXs in 516 IRL southern (*Sphoeroides nephelus*), checkered (*Sphoeroides testudineus*), and bandtail (*Sphoeroides spengleri*) puffer fish. During 36 months of monitoring, we detected STXs in skin, muscle, and viscera, with concentrations up to 22,104 μg STX equivalents (eq)/100 g tissue (action level, 80 μg STX eq/100 g tissue) in ovaries. Puffer fish tissues, clonal cultures, and natural bloom samples of *P. bahamense* from the IRL tested toxic in the MBA, RBA, MNCA, Ridascreen ELISA, and MIST Alert assay and positive for STX, dc-STX, and B1 toxin by HPLC and LC-MS. Skin mucus of IRL southern puffer fish captive for 1-year was highly toxic compared to Florida Gulf coast puffer fish. Therefore, we confirm puffer fish to be a hazardous reservoir of STXs in Florida’s marine waters and implicate the dinoflagellate *P. bahamense* as the putative toxin source.

**Conclusions:**

Associated with fatal paralytic shellfish poisoning (PSP) in the Pacific but not known to be toxic in the western Atlantic, *P. bahamense* is an emerging public health threat. We propose characterizing this food poisoning syndrome as saxitoxin puffer fish poisoning (SPFP) to distinguish it from PFP, which is traditionally associated with tetrodotoxin, and from PSP caused by STXs in shellfish.

Puffer fish poisoning (PFP) is usually caused by ingestion of tetrodotoxins (TTXs) found naturally in certain species of puffer fish (Halstead 1967; Mosher and Fuhrmann 1984). In Japan, 20–100 people die annually from PFP, in spite of stringent controls by authorities (Ogura 1971). TTXs can cause fatal human poisoning, which is similar to paralytic shellfish poisoning (PSP) caused by saxitoxins (STXs). PSP is caused by the consumption of toxic shellfish (Shumway 1990) and rarely by fish that have have become toxic after feeding on STX-producing microalgae (Maclean 1979). As well as TTXs, STXs have also been found in at least 12 marine and freshwater puffer fish species in Asia (Ahmed et al. 2001; Kodama et al. 1983; Kungsuwan et al. 1997; Nakamura et al. 1984; Nakashima et al. 2004; Sato et al. 1997, 2000; Zaman et al. 1997), but their bioorigin has not been identified.

TTXs are chemically distinct from STXs, but both neurotoxins produce similar symptoms in mammals because they act on site 1 of the voltage-dependent sodium channel, blocking the influx of sodium into excitable cells and restricting signal transmission along nerve and muscle membranes (Ahmed 1991). The symptoms of traditional PFP from TTXs and of PSP from STXs include tingling and numbness of the mouth, lips, tongue, face, and fingers; paralysis of the extremities; nausea; vomiting; ataxia; drowsiness; difficulty in speaking; progressively decreasing ventilatory efficiency; and finally in extreme cases, death by asphyxiation caused by respiratory paralysis (Ahmed 1991; Catterall 1985; Kao 1993).

PFP cases in Europe (Kao 1993) and Mexico (Nuñez-Vazquez et al. 2000) have occasionally been reported. In the United States, PFP has been associated with imports of puffer fish [Centers for Disease Control and Prevention (CDC) 1996]; rarely have fatalities occurred after the consumption of indigenous puffer fish. In Hawaii, white-spotted puffer fish, *Arothron hispidus*, were implicated in seven deaths (Ahmed 1991). Until 1974, seven PFP cases in Florida, outside of the Indian River Lagoon (IRL), were caused by the consumption of locally caught “blowfish” or puffer fish (Ahmed 1991; Benson 1956; Bigler 1999; Hemmert 1974; Mosher and Fuhrmann 1984). These cases included three fatalities, likely from TTX; for example, one woman died 45 min after consuming toxic liver from a checkered puffer fish (*Sphoeroides testudineus*) (Benson 1956). The toxins involved in the previous Florida PFP cases were not characterized, but because PFP is usually associated with TTX, investigators likely assumed that TTX was the cause (Benson 1956; Bigler 1999; Hemmert 1974). Tissues from Florida bandtail (*Sphoeroides spengleri*), checkered, and southern puffer fish (*Sphoeroides nephelus*) were found to be lethal in the mouse bioassay (MBA) (Burklew and Morton 1971; Lalone et al. 1963), but, again, the toxins were not determined.

Until January 2002 the harvest and consumption of puffer fish from the IRL was not a risk to public health. Since then (until May 2004), however, 28 PFP cases occurring in Florida (*n* = 21), New Jersey (*n* = 3), Virginia (*n* = 2), and New York (*n* = 2) caused by puffer fish originating from the IRL were reported (Bodager 2002; CDC 2002a, 2002b). Analyses of toxins from unidentified puffer fish fillet remnants from one of the early 2002 PFP cases in New Jersey revealed STXs (Quilliam et al. 2004), not TTXs, a distinction that alone could not be made on the basis of consumer symptoms or traditional screening methods (i.e., MBA).

During 2002–2004, all PFP cases were linked to puffer fish originating from the northern IRL and the Banana River on Florida’s east coast (Figure 1). Except for one case, where puffer fish were commercially harvested and reached a New Jersey fish market, puffer fish were caught recreationally [Bodager 2002; Florida Fish and Wildlife Conservation Commission (FWC) 2004]. In April 2002, state and federal officials issued health advisories, and the FWC banned puffer fish harvesting in the IRL, a ban that currently remains in effect. In New York on 14 October 2002, two PFP cases were caused from fish caught near Titusville, Florida, but frozen in March 2002 before the harvesting ban (Bodager D, personal communication). This case demonstrated the stability of toxins in puffer fish frozen for almost 9 months.

Because STXs had not previously been identified in Florida’s marine waters and their distribution, source, and origin were unknown in April 2002 (Abbott et al. 2003; Landsberg et al. 2002), we initiated an intensive survey of biota in the IRL. In this article we present a summary from 3 years of monitoring, as well as the first report of the putative toxin source.

## Materials and Methods

### Field collections

**Figure 1:**
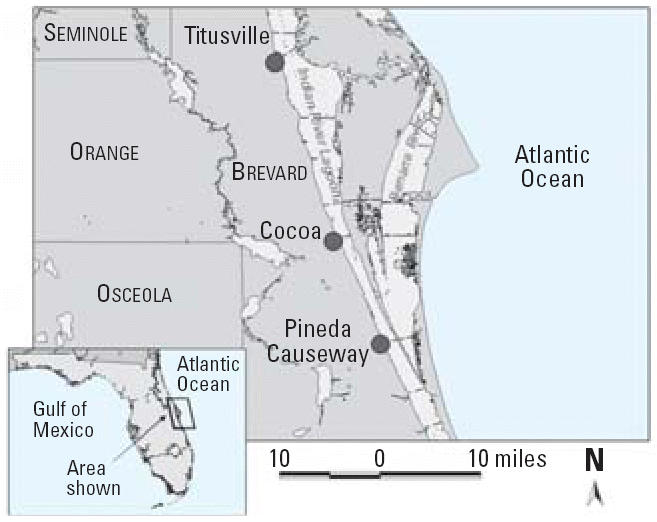
Map showing locations (circles) in the Indian River Lagoon, Florida, where toxic puffer fish in the SPFP incidents originated (FWC 2004). Sample collections of puffer fish and *Pyrodinium bahamense* were conducted throughout this area and further south to the St. Lucie River (not shown).

From April 2002 through April 2005, southern, checkered, and bandtail puffer fish (*n* = 516) were harvested via a range of fishing gear from the original source locations of the PFP incidents in the northern and central IRL (Figure 1). The fish were shipped biweekly or monthly on ice to the FWC’s Fish and Wildlife Research Institute (FWRI) or to the Food and Drug Administration’s (FDA) Center for Food Safety and Applied Nutrition Washington Seafood Laboratory and frozen in individual sealable plastic bags until required for toxicity testing.

Live phytoplankton samples were collected routinely with a 62-μm mesh plankton net at multiple locations along the IRL; also, a 1-L water bottle was used to directly sample a phytoplankton bloom. Water samples were transported to FWRI at ambient temperature.

### Live puffer fish

To determine if puffer fish maintained toxicity once they were removed from the putative toxin source, we kept puffer fish in captivity. We obtained southern puffer fish by rod and line or by seine net from the IRL near Titusville (Atlantic coast) (*n* = 2) and from Tampa Bay (Gulf coast), Florida (*n* = 2), and transported them live in ambient seawater to the wet laboratory at FWRI. Southern puffer fish were individually held in covered, 80-L aquaria in 25 psu (practical salinity units) artificial sea water (Instant Ocean; Aquarium Systems, Inc., Mentor, OH) and fed shrimp or squid that originated from non-toxic locations. We measured water quality daily and routinely carried out 30% water exchanges. After several weeks acclimation, we tested fish skin mucus bimonthly by lightly anesthetizing the fish [100 ppm tri-caine methanesulfonate (MS-222; Sandoz Pharmaceuticals Corp., Basel, Switzerland) in 4 L], placing the fish on a dissection board, and collecting the mucus on a preweighed 47-mm–diameter, glass-fiber filter (Whatman, Clifton, NJ) by gently rubbing the paper along both sides of the body.

### Fish care

We conducted research in compliance with the Animal Welfare Act and other federal statutes and regulations relating to animals and experiments involving animals. All fish were treated humanely and with regard for alleviation of suffering, according to the *Guide for Care and Use of Laboratory Animals* (Institute of Laboratory Animal Resources 1996).

### Preparation of tissues

Within several weeks of collection, we thawed frozen puffer fish, measured standard lengths and wet weights, and removed skin, liver, stomach, intestinal tract, muscle, and gonads.

### Pyrodinium bahamense *cultures*

We established 11 clonal nonaxenic cultures of *P. bahamense* from IRL samples, using the micropipette technique to isolate single cells. We maintained batch cultures in environmental chambers at near-ambient light and temperature conditions (35 microEinsteins/m^2^/sec, 25°C) and at salinities of 20–36 psu. Growth media consisted of filtered, autoclaved natural offshore seawater enriched to ES-DK (enriched natural seawater medium modified by D. Kulis) (Kulis D, personal communication; Kokinos and Anderson 1995) levels with the addition of 10^−7^ M selenium (as sodium selenite).

### Toxin detection

At various stages of this survey, we tested puffer fish tissues for STX bioactivity using the standard MBA, Ridascreen ELISA (R-Biopharm GmBH, Darmstadt, Germany), MIST Alert (Jellet Biotek, Dartmouth, Canada) PSP kit, and mouse neuroblastoma cytotoxicity assay (MNCA, Neuro-2A) and receptor-binding assay (RBA) [Association of Official Analytical Chemists (AOAC) 1990; Cembella et al. 2003; Jellett et al. 2002; Luckas et al. 2003; Powell and Doucette 1999; Ruberu et al. 2003; Usleber et al. 1991]. We also prepared selected samples for toxin characterization and confirmation by HPLC (Thermo Electron Corporation, San Jose, CA), with postcolumn oxidation and fluorescence detection and liquid chromatography-mass spectrometry (LC-MS) (Waters Corporation, Milford, MA) (Negri et al. 2003; Oshima 1995) using in-house FDA reference standards. We split tissue samples for interlaboratory calibrations and then extracted them by one of two methods. For bioactivity assays, tissue samples were homogenized and weighed (wet weight) into glass test tubes. Samples were extracted using 0.1 N HCl, adjusted to pH 2.5–4, boiled for 5 min in a boiling water bath, centrifuged at 3,000 × *g* for 10 min, and the supernatant retained for toxin testing. For toxin characterization by HPLC and LC-MS, tissue splits were extracted with 0.1 M aqueous acetic acid, centrifuged, and the supernatants filtered (0.22 μm).

After the initial 2002 saxitoxin puffer fish poisoning (SPFP) events, 11 southern puffer fish were divided into the tissue compartments (listed above), and tissue samples were extracted by boiling in 0.1 N HCl (Washington Seafood Laboratory) and analyzed for toxic activity using three independent methods. MBAs were performed at the Washington Seafood Laboratory; MNCAs were performed at the FDA Gulf Coast Seafood Laboratory; and RBAs were performed at the National Oceanic and Atmospheric Administration National Ocean Service Center for Coastal Environmental Health and Biomolecular Research.

Clonal cultures and natural bloom samples of *P. bahamense* were filtered onto 25-mm glass-fiber filters (Whatman) or centrifuged at 3,000 × *g* for 5 min and then extracted. Puffer fish mucus or *Pyrodinium* samples on filters were homogenized in 0.1 N HCl using a ground-glass tissue grinder and treated as above. *Pyrodinium* extracts were tested for toxicity by ELISA and MBA and characterized for toxin profile using HPLC and LC-MS. Puffer fish mucus was tested for toxicity by ELISA.

### Electron microscopy

We prepared natural field samples or clonal cultures of *P. bahamense* for the scanning electron microscope (SEM) using standard fixation methods (Truby 1997). *Pyrodinium* samples were added to unacidified Lugol’s at a dilution of 1:100 in suspension, collected onto a 5-μm polycarbonate filter, secondarily fixed with 4% paraformaldehyde for 20 min, washed with water, dehydrated in an ethanol series followed by a freon series, critical-point-dried using carbon dioxide, mounted onto aluminum stubs using carbon adhesive tape, sputter-coated with gold/palladium, and photographed with a Cambridge Stereoscan 240 SEM (Cambridge Instruments, Cambridge, UK).

## Results

### Puffer fish toxin analyses

During 36 months of continuous monitoring after the initial SPFP events, STXs were routinely detected in the skin, muscle, viscera, and gonads of 516 puffer fish (southern, *n* = 402; checkered, *n* = 105; bandtail, *n* = 9), which tested toxic in MBA, RBA, Ridascreen ELISA, and MIST Alert assays. By ELISA, maximum STX levels in the muscle fillet were well above the action level [80 μg STX equivalents (eq)/100 g tissue] in southern puffer fish (maximum, 14,571 μg STX eq/100 g tissue, mean = 938.4) and just over the action limit in bandtail (maximum, 364.5 μg STX eq/100 g tissue; mean, 121.7) and checkered puffer fish (maximum, 104.3 μg STX eq/100 g tissue; mean, 6.9) (Table 1). Maximum STX concentrations in the liver of southern and checkered puffer fish were 1,443 and 51.1 μg STX eq/100 g tissue, respectively. The highest tissue concentration, 22,104 μg STX eq/100 g tissue, was measured in the ovaries of a southern puffer fish (data not shown).

All three assays (MBA, MNCA, and RBA) confirmed elevated concentrations of toxic activity in the muscle compared to the liver (5- to 20-fold) of 11 southern puffer fish (Table 2). By MBA, MNCA, and RBA, ranges of STX concentrations in muscle were 197–5,264 (mean ± SD, 2,302.3 ± 1,539.3), 120–2,294 (957.7 ± 659.5), and 198–6,091 (2,439 ± 1,995.3) μg STX eq/100 g tissue, respectively. By MBA, MNCA, and RBA, STX concentrations in liver were 83–1,034 (mean ± SD, 282.6 ± 261.5), 60–420 (169.1 ± 106.2) and 16–711 (223.1 ± 186.5) μg STX eq/100 g tissue, respectively (Table 2).

Skin mucus of IRL southern puffer fish held captive for 1 year was highly toxic (2,407–9,039 μg STX eq/100 g) compared with that of Florida Gulf Coast southern puffer fish (6.25–140 μg STX eq/100 g). Over a period of at least 6 months, STX levels in the IRL southern puffer fish fluctuated but remained at highly toxic concentrations.

**Figure 2:**
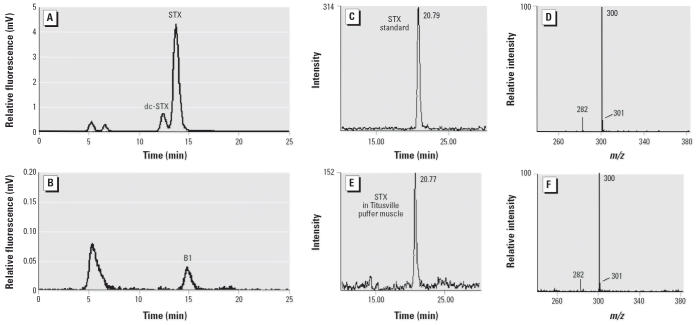
Toxin analysis of southern puffer fish muscle. HPLC chromatograms showing (*A*) dc-STX (7%) and STX (92%), and (*B*) B1 (1%). LC-MS ion chromatograms (*C*, *E*) and mass spectra (*D*, *F*) of STX in reference standard (*C*, *D*) and Titusville puffer fish muscle (*E*, *F*).

Toxin profiles in unconsumed puffer fish fillets (*n* = 4) from a 2004 PFP event were confirmed by HPLC (Figure 2A,B) and LC-MS (Figure 2C–F) to be STX (92.4% ± 3.1), decarbamoyl saxitoxin (dcSTX; 6.9% ± 2.4), and N-sulfocarbamoyl B1 toxin (B1; 0.7% ± 0.7) as originally found in a 2002 PFP case (Quilliam et al. 2004).

**Figure 3:**
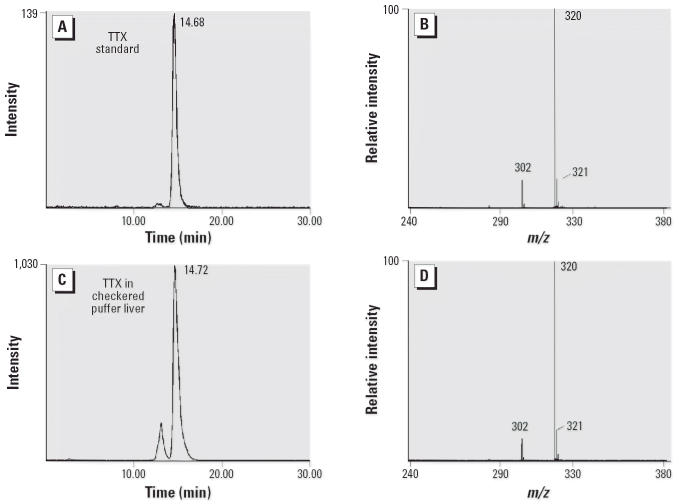
LC-MS ion chromatograms (*A*, *C*) and mass spectra (*B*, *D*) of TTX in reference standard (*A*, *B*) and checkered puffer fish liver (*C*, *D*).

We also detected TTX (quantified by MBA and confirmed by LC-MS) in IRL checkered puffer fish (*n* = 3) at concentrations of 1,553 ± 919 and 53,700 ± 19,212 μg TTX/100 g in the muscle and liver, respectively (Figure 3A–D).

### Pyrodinium bahamense *toxin analyses.*

**Figure 4:**
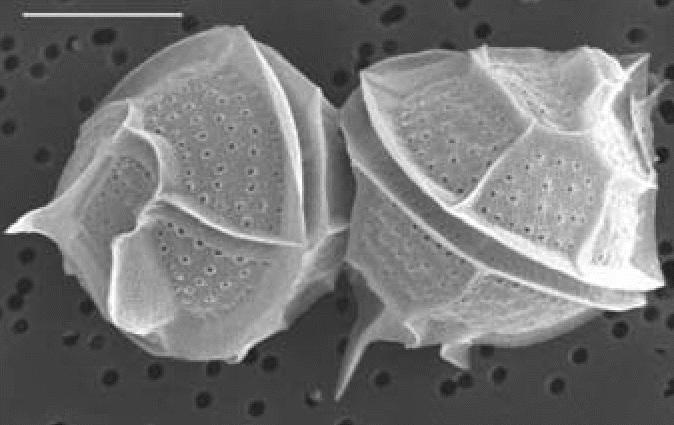
Scanning electron micrograph of two *P. bahamense* cells isolated from the IRL (FWC 2004). Left cell, posterior-lateral view; right cell, dorsal view. Bar = 20 μm.

**Figure 5:**
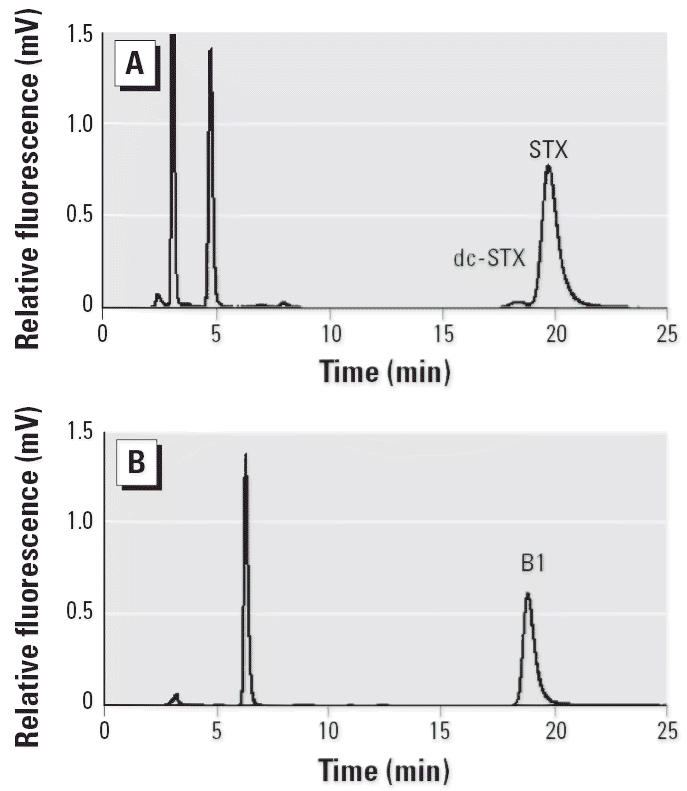
Toxin analysis of *P. bahamense* by HPLC chromatograms showing (*A*) dc-STX (1%) and STX (26%), and (*B*) B1 (73%).

All clonal cultures (*n* = 11) and natural bloom samples (*n* = 2) (> 3 million cells/L) of *P. bahamense* (Figure 4) obtained from the IRL tested positive for STX by HPLC, LC-MS, Ridascreen ELISA, MIST Alert, and RBA assays. Toxin concentrations for *P. bahamense* isolates (*n* = 11) ranged from 1.68 to 25.57 pg STX eq/cell (as determined by ELISA). Further analysis of five of these isolates using HPLC determined that the toxin profile was composed of B1 (91.1% ± 2.2, mean ± SD) and STX (8.9% ± 2.2), with integrated toxicity values ranging from 2.02 to 12.74 pg STX eq/cell. The HPLC toxin profile of a 2002 bloom sample at 3.28 pg STX eq/cell was composed of STX (26%), B1 (73%), and dcSTX (1%) (Figure 5A,B).

## Discussion

PFP cases have been associated with STXs in Asia (Ahmed et al. 2001), but the IRL incidents are the first in which STX poisoning has been confirmed in puffer fish originating in the United States (Quilliam et al. 2004). The high and low concentrations of STXs and TTX, respectively, in the muscle of IRL puffer fish are similar to those found in Philippine (Sato et al. 2000) and Japanese (Kodama et al. 1984) puffer fish, although in the latter, visceral toxicity from TTX is high and fish-poisoning incidents usually occur after consumption of fillet(s) contaminated due to improper preparation. Unlike the tissue distribution of TTXs reported previously in various puffer fish species (Kodama et al. 1984), STXs in IRL southern puffer fish have been consistently much higher in the muscle than in the liver and, in many individual fish, were more than two orders of magnitude above the action limit. Therefore, even careful preparation of IRL puffer fish fillets would not prevent intoxication in consumers. Interestingly, the confirmation of extremely high concentrations of TTX in the liver of checkered puffer fish suggests that the earlier-reported fatality from the consumption of this species in south Florida (Benson 1956) was likely caused by this toxin and not STX.

The MBA, the traditional screening method for PFP, does not distinguish between STXs and TTXs. New reports in Asia (Ahmed et al. 2001; Nakashima et al. 2004; Sato et al. 2000) have found both toxin groups co-occurring in puffer fish species previously thought to contain only TTX. Both our results and these reports suggest that STXs in puffer fish may be more widespread than previously thought; therefore, comprehensive analytical assessments of PFP incidents are needed to distinguish TTX from STX. We propose that the food-poisoning syndrome caused by intoxication from STX exposure from fish should be characterized as SPFP to distinguish it from PFP, which is caused by—but not always verified to be from—TTX, and to distinguish SPFP from PSP associated with STXs in shellfish.

In a 1960s toxicity study of IRL southern puffer fish [erroneously identified by Lalone et al. (1963) as northern puffer fish, *Sphoeroides maculatus*, which are not found in the IRL and occur only as far south as Jacksonville, FL (Shipp and Yerger 1969a, 1969b; Tremain and Adams, 1995)], muscle was demonstrated to be toxic to mice by intraperitoneal injection. However, the toxins in these puffer fish samples were not characterized. Of the tissues investigated in that study, including skin, liver, muscle, and testes or ovary, the muscle was the most lethal to mice, similar to the pattern seen today. Although this anecdotal evidence suggests that southern puffer fish may have been mildly toxic from STX in the IRL for the past 45 years, there has been no indication that toxin levels were even close to the order of magnitude observed since 2002 nor was the FDOH informed of any poisoning incidents from this area prior to this time.

Globally, human food-poisoning incidents from STX exposure are usually caused by toxic marine shellfish (Kao 1993) that filter-feed on STX-producing microalgae. PSP can be fatal (Kao 1993), but the successful implementation of programs monitoring STX-producing microalgae and STXs in shellfish has helped minimize the risk of toxin exposure to humans. In marine waters, PSP is caused by toxic dinoflagellates, where STXs are produced by more temperate *Alexandrium* species and *Gymnodinium catenatum* and by tropical *Pyrodinium bahamense* var. *compressum* (Kao 1993). PSP in the United States has been limited to New England and the Pacific West Coast, including Alaska, and has only been associated with STXs produced by temperate *Alexandrium* spp. in these areas (Gessner 2000).

The epidemiology of PSP incidents is related to the global distribution of the various STX-producing species and their toxigenic strains. PSP outbreaks due to *P. bahamense* have caused more fatalities than any other microalgal species known (Usup and Azanza 1998). In 1987, PSP associated with *P. bahamense* var. *compressum* in Champerico, Guatemala, hospitalized at least 187 individuals and resulted in 26 fatalities (Rodrigue et al. 1990). Before 1996, 1,768 cases of PSP with 107 deaths had been reported in the Philippines, mostly attributable to *P. bahamense* var. *compressum* (Babaran et al. 1998). These fatalities were largely due to the sudden appearance of *P. bahamense* in areas previously unknown to contain toxic species, because monitoring activities were not in place or because hospital facilities had not treated people in these previously unaffected areas (Kao 1993).

In the present study we confirm unequivocally that puffer fish are a primary reservoir of STXs in marine waters in Florida, and we implicate for the first time the tropical western Atlantic dinoflagellate *P. bahamense* as the source of toxicity. We found the STX profile of *P. bahamense* isolates from Florida to be similar to, but proportionately different from, the toxin profile of southern puffer fish fillet (Etheridge et al. 2006; Quilliam et al. 2004), and we identified *P. bahamense* as the putative source of the STXs. Confirmatory toxin-transfer studies from *Pyrodinium* via shellfish to puffer fish are in progress. Although many temperate marine *Alexandrium* species, *Gymnodinium catenatum*, and a few freshwater cyanobacteria species produce STXs (Kodama 2000), these organisms have not been found in the IRL.

In addition to the Caribbean and Gulf coasts of Mexico, bioluminescent *P. bahamense* blooms are found only along Florida’s Atlantic and Gulf coasts (Badylak et al. 2004; Phlips et al. 2004; Steidinger et al. 1980). However, until the IRL SPFP incidents, the Atlantic/Caribbean *P. bahamense* var. *bahamense* was not known to be toxic (Steidinger et al. 1980), unlike the Pacific *P. bahamense* var. *compressum* found in Asia and the Pacific Coast of Central America (Rodrigue et al. 1990; Usup and Azanza 1998; Vargas-Montero and Freer 2004). The Atlantic *P. bahamense* var. *bahamense* was separated from the Pacific *P. bahamense* var. *compressum* based on morphologic criteria and evident lack of toxicity in the former variety (Steidinger et al. 1980). Based on our initial findings, we are testing the hypothesis that this varietal distinction may no longer be valid and that *P. bahamense* is all one species.

Florida has many toxigenic marine algal species, but none were known to produce STXs (Steidinger et al. 1999). It is conceivable that STXs might have appeared in the IRL because of one of several scenarios: *a*) toxigenic populations of *Pyrodinium* have been introduced; *b*) ecologic conditions have changed and have induced toxicity in a variety that was previously nontoxic; *c*) toxic *Pyrodinium* was present but produced toxins at undetectable concentrations; or *d*) ecologic conditions have changed and increased the food-web exposure of susceptible biota to toxins. We believe that *c* is the most likely scenario. In the IRL there is a history of *Pyrodinium* (Badylak et al. 2004; Phlips et al. 2004), and as mentioned previously, there is a historical precedent for low-level toxicity in puffer fish.

In the past few years, the northern IRL has experienced a number of unusual events: dolphin, manatee, fish, and horseshoe crab mortalities; increased tumor incidence in hard clams; diseased shrimp; and reductions in the natural recruitment of and increases in the hatchery losses of hard clams (Bossart et al. 2003; Landsberg et al. 2002; Landsberg and Kiryu 2005). To what extent, if at all, these events are linked to the emerging issue of toxic *P. bahamense* blooms remains undetermined. The significant risk of SPFP and PSP from saxitoxins in the IRL has been assessed and management plans implemented accordingly. Thus far, routine monitoring by Florida state agencies has determined that STX levels in shellfish, principally hard clams (*Mercenaria* spp.), are not a significant risk to public health (Landsberg et al. 2005). The extreme toxicity of puffer fish fillet, well above the action level, emphasizes the danger that puffer fish pose to the public and supports the permanent ban on their harvest in this area (FWC 2004).

The widespread implications for public health incidents from the tropical western Atlantic *P. bahamense* remain unknown. Public health officials and natural resource managers should be aware of these new findings and remain vigilant to examine any potential association between the co-occurrence of this species throughout its range and the appearance of toxic food-poisoning incidents.

## Figures and Tables

**Table 1:** Comparison of saxitoxin concentrations (μg STX eq/100 g tissue) in muscle and liver of IRL puffer fish species by ELISA.

	Muscle			Liver		
Puffer fish species	No.	Mean ± SD	Maximum	No.	Mean ± SD	Maximum
Southern	402	938.4 ± 1,418	14,571	55	265.6 ± 393	1,443
Checkered	105	6.9 ± 11.4	104.3	3	20.3 ± 27.1	51.1
Bandtail	9	121.7 ± 117.9	364.5	0	—	—

**Table 2:** Comparison of saxitoxin-like activity levels (μg STX dihydrochloride eq/100 g tissue) by LC-MS in muscle and liver of southern puffer fish (*S. nephelus*) collected from the IRL after the first SPFP cases in 2002.

	MBA			RBA			MNCA		
Fish	Muscle	Liver	Fold diff	Muscle	Liver	Fold diff	Muscle	Liver	Fold diff
1	5,264	1,034	5.1	4,136	711	5.8	2,294	420	5.5
2	4,697	376	12.5	6,091	304	20.0	1,230	280	4.4
3	2,986	242	12.3	2,433	280	8.7	1,947	160	12.2
4	2,804	203	13.8	1,423	147	9.7	1,100	120	9.2
5	2,564	149	17.2	5,253	297	17.7	844	110	7.7
6	2,153	135	15.9	2,911	173	16.8	790	240	3.3
7	1,970	263	7.5	2,257	142	15.9	750	150	5.0
8	1,216	254	4.8	805	154	5.2	350	140	2.5
9	1,098	221	5.0	1,089	180	6.1	480	110	4.4
10	376	83	4.5	198	16	12.4	630	70	9.0
11	197	149	1.3	231	50	4.6	120	60	2.0

Fold diff indicates fold difference of muscle compared with liver.
